# Long-term mortality of patients with sepsis is worse than that of non-septic

**DOI:** 10.1186/2197-425X-3-S1-A879

**Published:** 2015-10-01

**Authors:** G Friedman, L Biason, C Teixeira

**Affiliations:** PPG Ciências Pneumológicas, Universidade Federal do Rio Grande do Sul, Porto Alegre, Brazil; Clínica Médica, Universidade Federal de Ciências da Saúde de Porto Alegre, Porto Alegre, Brazil

## Introduction

Long-term outcomes of septic patients are poorly studied.

## Objectives

Our goal is to determine the rate of mortality and quality of life (QOL) after 2 years of patients with severe sepsis and compare to non-septic patients.

## Methods

Prospective cohort of 1219 patients of 2 mixed ICU. Patients were followed for 24 months after ICU discharge. We evaluated mortality and quality of life by Karnofsky scale and ADL (Activities Daily Living).

## Results

Septic patients (n = 442) had higher mortality rate than non-septic (n = 777) in the ICU (41.6 vs. 13.6%, p < 0.0001), and after two years (74.8 vs. 42.3%, p < 0.0001) - Figure. The QOL of septic patients was lower than the non-septic at ICU admission and after two years. (1) Karnofsky pre-ICU 84 ± 11 vs. 88 ± 16 (p = 0.02), after 2 years: 76 ± 19 vs. 82 ± 17 (p = 0,007); ADL pré-ICU 26 ± 10 vs. 28 ± 7 (p = 0.014), after 2 years 22 ± 11 vs. 25 ± 10 (p = 0.011).

## Conclusions

Patients suffering an episode of severe sepsis have increased mortality as compared with non-septic.

**Figure 1:**
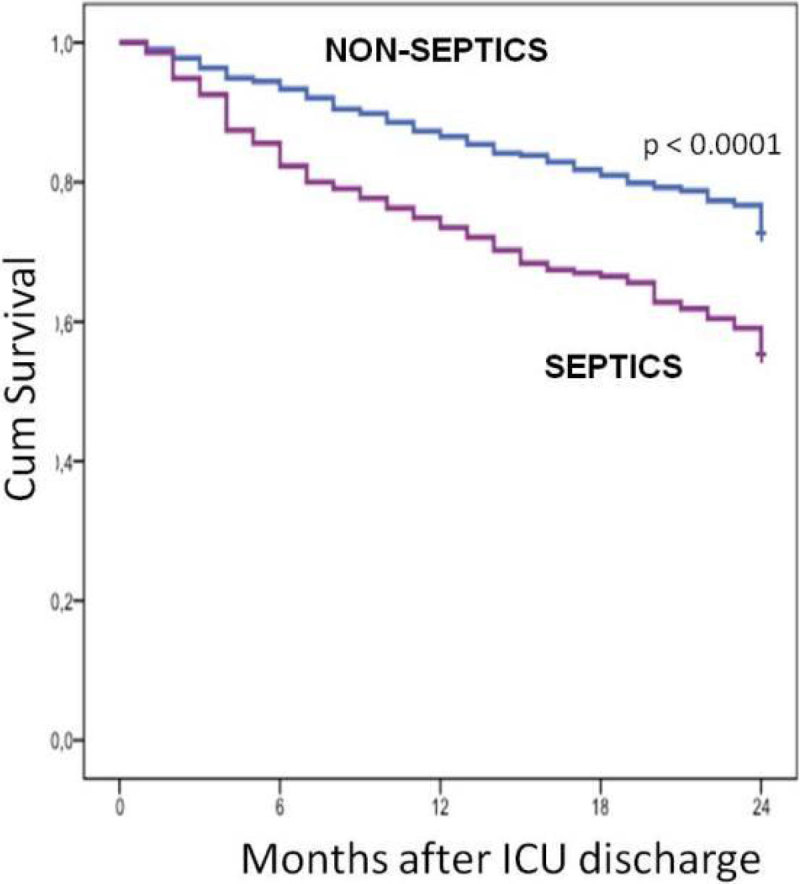
Figure 1
